# The song remains the same although the instruments are changing: complications following selective non-operative management of blunt spleen trauma: a retrospective review of patients at a level I trauma centre from 1996 to 2007

**DOI:** 10.1186/1752-2897-6-4

**Published:** 2012-03-13

**Authors:** Aisling A Clancy, Corina Tiruta, Dianne Ashman, Chad G Ball, Andrew W Kirkpatrick

**Affiliations:** 1 Queen's University School of Medicine, Kingston, ON, Canada; 2 University of Ottawa, Ottawa, ON, Canada; 3 Regional Trauma Services, Calgary, AB, Canada; 4 Departments of Surgery, Calgary, AB, Canada; 5 Critical Care Medicine, Calgary, AB, Canada; 6 Departments of Surgery and Critical Care Medicine, Foothills Medical Centre, Calgary, AB, Canada; 7 Regional Trauma Services, 1403 29 St NW, Calgary, AB T2N 2 T9, Canada

**Keywords:** Splenic injury, Surgery, Resuscitation, Diagnostic imaging, Angiography

## Abstract

**Background:**

Despite a widespread shift to selective non-operative management (SNOM) for blunt splenic trauma, there remains uncertainty regarding the role of adjuncts such as interventional radiological techniques, the need for follow-up imaging, and the incidence of long-term complications. We evaluated the success of SNOM (including splenic artery embolization, SAE) for the management of blunt splenic injuries in severely injured patients.

**Methods:**

Retrospective review (1996-2007) of the Alberta Trauma Registry and health records for blunt splenic trauma patients, aged 18 and older, with injury severity scores of 12 or greater, admitted to the Foothills Medical Centre.

**Results:**

Among 538 eligible patients, 150 (26%) underwent early operative intervention. The proportion of patients managed by SNOM rose from 50 to 78% over the study period, with an overall success rate of SNOM of 87%, while injury acuity remained unchanged over time. Among SNOM failures, 65% underwent surgery within 24 hours of admission. Splenic arterial embolization (SAE) was used in only 7% of patients managed non-operatively, although at least 21% of failed SNOM had contrast extravasation potentially amenable to SAE. Among Calgary residents undergoing SNOM, hospital readmission within six months was required in three (2%), all of whom who required emergent intervention (splenectomy 2, SAE 1) and in whom none had post-discharge follow-up imaging. Overall, the use of post-discharge follow-up CT imaging was low following SNOM (10%), and thus no CT images identified occult hemorrhage or pseudoaneurysm. We observed seven cases of delayed splenic rupture in our population which occurred from five days to two months following initial injury. Three of these occurred in the post-discharge period requiring readmission and intervention.

**Conclusions:**

SNOM was the initial treatment strategy for most patients with blunt splenic trauma with 13% requiring subsequent operative intervention intended for the spleen. Cases of delayed splenic rupture occurred up to two months following initial injury. The low use of both follow-up imaging and SAE make assessment of the utility of these adjuncts difficult and adherence to formalized protocols will be required to fully assess the benefit of multi-modality management strategies.

## Background

Over the last forty years, selective non-operative management (SNOM) has replaced emergent splenectomy in hemodynamically stable patients [1-3]. This approach relies upon accurate diagnostic imaging (predominantly computed tomography (CT)) and may be supplemented by angiography. All imaging technologies, including CT, have undergone concurrent improvements in image fidelity and information content. The improved utilization of percutaneous techniques to control hemorrhage has also encouraged SNOM [4-6]. In general, SNOM of splenic injury has numerous potential benefits including fewer blood transfusions, shorter hospital stays, avoidance of long-term infectious complications and lower surgical costs [7,8].

Despite these successes, failure of SNOM can be a life-threatening event, increases resource use and hospital length of stay, and makes the use of spleen salvaging techniques less likely within the operating room. Failure of SNOM has been attributed to ongoing or spontaneous hemorrhage, missed injury, delayed splenic rupture or development of a splenic artery pseudoaneurysm. Some authors believe that failures are related to splenic arterial injuries that are either missed (due to limited size) or subsequently develop after initial imaging following lysis of clotted blood at the site of injury [9]. Renewed efforts to continually improve the outcomes of SNOM have included routine post-admission diagnostic imaging and/or selective angiography/angio-interventional procedures [4,5,10-12]. Despite these recommendations, the value of imaging studies in long-term follow-up of patients undergoing SNOM is unclear [13]. As a result, there is extensive variability in both the frequency and timing of follow-up of imaging [11,14]. Although most failures of SNOM occur within the first 72 hours following admission [2,15], delayed splenic rupture has also been observed in patients up to months following injury [16-18]. Further, a published analysis of readmissions after SNOM of splenic injury revealed a 1.4% rate of readmission for splenectomy in the 180 days post-discharge, suggesting a need for improved outpatient management and follow-up [19].

The primary goal of this study was to evaluate the success rates of various management strategies (observation, immediate splenectomy or splenorrhaphy, splenic vessel embolization) and to review the characteristics and outcomes of patients failing SNOM during hospitalization, as well as in the post-discharge period. We examined the clinical application and complications resulting from use of adjunct Splenic Artery Angiography (SAA) in our institution both in Operative Management (OM) and SNOM patients. Additionally, we aimed to evaluate the use and effectiveness of follow-up imaging (both in-hospital and post-discharge) in our population.

## Methods

### Setting

The Alberta Health Services Calgary Zone is a fully integrated, publicly funded health system that provides virtually all medical and surgical care to the residents of the city of Calgary and a large surrounding area (population ~ 1.2 million). Within the Calgary Zone, adult trauma services are regionalized to the Foothills Medical Centre (FMC). This is the only Level One trauma center in Southern Alberta, the south-eastern parts of Saskatchewan and the south-west of British Columbia, and as so functions as the referral center of a large inclusive trauma system.

### Data source

A retrospective review of all trauma patients with blunt splenic injuries admitted to Foothills Medical Centre (1996-2007) was performed. Patients were identified using the Alberta Trauma Registry, which includes data on all patients with an injury severity score (ISS) of 12 or greater who are admitted to hospital or who die in the trauma centre's Emergency Department (ED). Registry records contain information on patient demographics, injury mechanism, location and severity of all injuries, as well as therapeutic interventions and hospital disposition. The study was approved by the Conjoint Health Research Ethics Board at the University of Calgary (ID 21800) and is in compliance with the Helsinki Declaration.

### Eligibility

We included patients 18 years or older, with ISS scores of 12 or higher, admitted between January 1, 1996 and December 31, 2007 as a result of blunt trauma that included injury to the spleen. The registry was searched for all records with a code of "blunt" for type of injury, and an Abbreviated Injury Scale (AIS) predot code from 544010 to 544240 or 544299 [20]. Patients were excluded if they died in the emergency department, had undergone a laparotomy prior to arrival, or were transferred to the trauma centre for follow-up of orthopaedic or head injuries after receiving their definitive trauma management at another institution.

### Data collection

The trauma registry was used to determine age, sex, external cause of injury (coded using the International Classification of Diseases, 9^th ^Revision, Clinical Modification) [21], first recorded heart rate, first recorded systolic blood pressure, Glasgow Coma Scale (GCS, if quantifiable), abbreviated injury scale (AIS) scores and ISS. A detailed medical record review was also performed for all eligible patients. Various clinical characteristics were documented including pre-existing comorbidities, blood product and crystalloid requirements, first and lowest documented hemoglobin, diagnostic imaging results, resuscitation and diagnostic procedures performed in the emergency department, surgeries and any associated complications.

To assess long-term complications associated with splenic injury, we attempted to ascertain if any Calgary residents were re-admitted to hospital within the Calgary Health Region in the six-month period following hospital discharge. This was done by querying Clinibase (computerized admitting, discharge and transfer system in the Calgary Health Region, Logibec Groupe Informatique Ltd, Montreal, QC). For the same patient population, we also attempted to identify abdominal imaging (MRI, CT, US or angiography) completed in the same time period by querying our Radiology Information System (QuadRIS, version 6.3, ADAC HealthCare Information Systems, Houston, TX).

### Outcomes

Operative management (OM) was defined as splenectomy, splenorrhaphy or laparotomy within four hours of arrival to the trauma centre. All other patients (including those who received SAE) were considered to be managed non-operatively. In-hospital failure of SNOM was defined as any surgical intervention (excluding SAE) for the splenic injury that occurred more than four hours after arrival as previously defined by Cocanour and colleagues [18]. We also included exploratory laparotomies without any therapy for a specific injury in the failure of SNOM group assuming the intention was to deal with a splenic injury, while we did not include therapeutic laparotomies to repair/manage other specific injuries other than splenic trauma in the failure of SNOM group. Delayed splenic rupture was defined as failure of SNOM more than 48 hours after initial injury, consistent with the definition of delayed splenic complications defined by Cocanour [9]. Post-discharge failure of SNOM was defined as any surgical intervention (excluding SAE) within six months of hospital discharge. Complications of SNOM (aside from overt failure requiring operative intervention) included infection or sepsis, splenic abscess, post-traumatic splenic artery pseudoaneurysm and splenic infarct.

### Data analysis

Simple descriptive statistics were used to summarize all study variables (means and standard errors for normally distributed variables; medians and interquartile ranges for non-normally distributed variables). For each study year, the proportion of patients initially treated with SNOM was determined, and median ISS and mortality were calculated by initial management strategy (OM vs. SNOM). The characteristics of patients treated with OM versus SNOM and the proportion of SNOM patients who received adjunct SAE therapy were also assessed. All analyses were done using Stata (version 9, College Stn, Texas).

## Results

### Operative versus non-operative management over time

Over the study period, 619 patients with blunt splenic trauma were identified in the registry. Eighty-one were excluded (23 without a spleen injury, 8 younger than 18 years, 7 deceased in the emergency department, 17 with abdominal surgery prior to arrival, 10 late transfers following definitive trauma care elsewhere, and 16 missing medical records), leaving 538 for analysis. The median ISS was 27 (IQR 19-34) for all patients, 34 (IQR 25-43) for the operative group, and 24 (IQR 17-32) for the SNOM cohort.

The use of SNOM increased over time, ranging from about 50% in 1996 to nearly 80% in 2007. Overall, those selected for OM were more severely injured, had lower arrival systolic blood pressure (SBP); were more likely to be hemodynamically unstable and required more crystalloid and packed red blood cells (RBCs) within the first four hours of arrival. Mortality among patients managed operatively was variable over time, ranging from 13% to 40% with no appreciable trend. Mortality among SNOM patients increased slightly over time, from 0% in 1996 to 3% in 2007. None of these deaths were directly attributed to splenic injury.

Patient demographic, clinical and injury characteristics were analyzed and are available in Additional file 1. Approximately half of all patients were Calgary residents, and 38% were transferred to the trauma centre following initial triage at another facility. Patients ranged in age from 18 to 89 years (median 34 years, IQR 23-49). As expected with major trauma, males and individuals 18-35 years of age accounted for about 75% and 50%, respectively, of all cases. The age and sex distributions were similar for OM and SNOM. The prevalence of pre-existing conditions was also similar across groups, with one or more preexisting conditions documented for about 25% of all patients. Hypertension, type 2 diabetes mellitus and asthma were the most commonly listed comorbidities (data not shown). OM patients had higher median ISS and a greater proportion sustained severe head and abdominal injuries (i.e., maximum abbreviated injury scores, MAIS > 3).

Of the patients who were admitted more than 24 hours following the injury event three patients had a missed splenic injury and were discharged after a first ED visit or admission, eight patients were transferred a from a rural centre and three patients had a delayed self-presentation to the ED. None of the patients with missed spleen injuries received abdominal imaging on their initial admission or visit to the ED and all presented with symptoms following their initial admission/ED visit. Two patients with missed injuries required subsequent splenectomy and one was managed by SNOM.

### Failure of Selective Non-operative management and readmission of SNOM patients

The overall success rate of SNOM (i.e. patients did not require abdominal surgery for the spleen during admission) was 87%. This correlated with a progressive use of SNOM from 50% to 78% over the course of the review. Among those selected for non-operative management, 60 (16%) went on to have a surgical procedure; which consisted of consisted of splenectomy (9%, 35 patients), splenorrhaphy (2%, 7 patients), or laparotomy without splenic intervention (5% [exploratory 2%, 8 patients, and non-splenic therapeutic 3%, 10 patients]). Those who failed non-operative management, had a higher mortality, received more blood and crystalloid fluid within the first four hours, were slightly older, with higher median ISS scores, lower blood pressure, and stayed twice as long in hospital if they survived compared to those who had successful SNOM. Of this group who failed SNOM (n = 53), 33 (63%) arrived at hospital within four hours of injury with only three (6%) arriving more than 24 hours after injury.

Overall, 39% (210 patients) of the study group underwent abdominal surgery at some point during their acute care stay. Twenty-five percent (38 patients) of the operative group underwent laparotomy for interventions not involving the spleen. Ultimately 29% (154 patients) of all study patients underwent splenorrhaphy or total splenectomy. The median time to surgery (exploratory laparotomy, splenorrhaphy or total splenectomy) for those who failed was 11.2 hours (IQR 6.7-47.1). Of these, 16 (30%) required OM in less than 8 hours, 12 (23%) between 8 to 12 hours, 9 (17%) between 12-24 hours, and only 16 (30%) more than 24 hours after admission. The initial CT scans of patients who failed SNOM finding that 21 patients (40% of SNOM failures) had splenic injury grades of 4-5. Twenty-two patients (41%) who failed SNOM had splenic injuries grades of 1 to 3 and the splenic injury grade was undetermined in 10 (19%) of SNOM failures (ie. initial CT not available for grading). The charts and imaging of patients who failed SNOM were reviewed in more detail.

**Figure 1:**
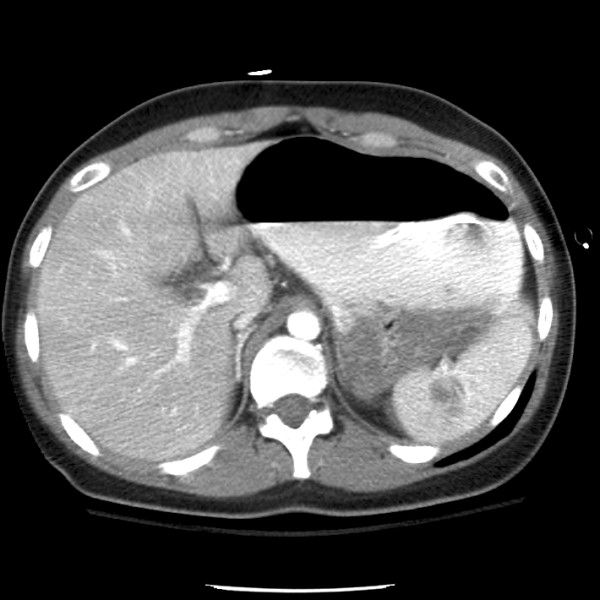
**Abdominal CT scan of parenchymal splenic laceration without obvious vascular injury at 01:16 am**.

**Figure 2:**
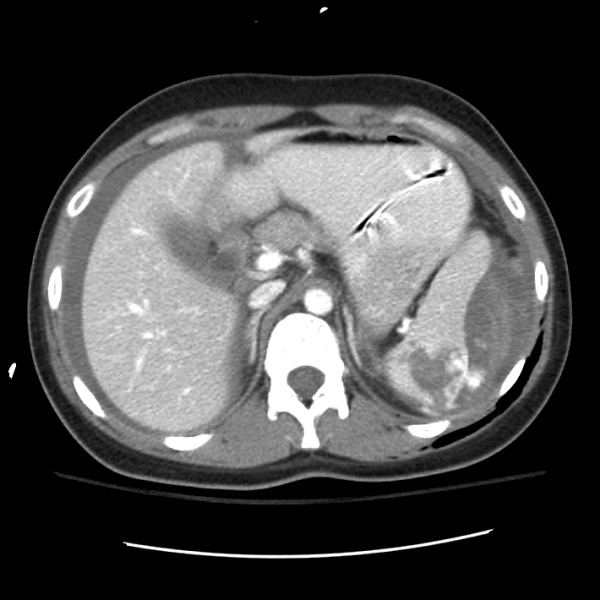
**Abdominal CT scan of same patient revealing active contrast extravasation at 08:13 am**.

Six patients had an active blush or suspected pseudoaneurysm on the initial CT scan upon which SNOM would presumably be based. Of the six patients for whom SNOM was attempted without addressing the blush or early pseudoaneurysm, one may have been related to an inadvertent delay to OM, with the remaining five cases occurring early in the series (four cases from 2001 and one case from 2003). Five patients who failed SNOM also developed active extravasation present on follow-up CT scans which were done nine hours (Figures 1 and 2), two, seven, nine, and ten days after admission. Overall, 11 of 53 (21%) failures of SNOM revealed acute vascular extravasation. A more detailed comparison between those patients treated successfully compared with those who failed SNOM is also presented in Table 1.

**Table 1:** Characteristics of non-operatively managed patients compared by success or failure of non-operative management

Variable	Failure of NOM N = 53 (14) n (%)	Successful NOMN = 335 (86) n (%)
**Sex**		

Male	39 (74)	239 (71)

Female	14 (26)	96 (29)

**Time from injury to hospital arrival (hours)**		

Less than 1 hour	20 (38)	85 (25)

1 to 4 hours	13 (25)	116 (35)

4 to 24 hours	11 (21)	98 (29)

More than 24 hours	3 (6)	9 (3)

Not documented	6 (11)	27 (8)

**Received pRBCs^g ^in the first 4 hours**		

Yes	22(42)	43(13)

No	30(56)	286(85)

Not documented	1 (2)	6 (2)

**Received pRBCs^g ^during hospital admission**		

Yes	40(75)	109(33)

No	13(25)	226(67)

**Died during hospital admission**	3(7)	11 (3)

**Median age (years) (IQR^b^)**	38 (25-52)	34 (23-49)

**Median Injury Severity Score (IQR^b^)**	29 (20-42)	24 (17-29)

**Mean systolic BP^e ^(mmHg) on hospital arrival (SD^d^)**	114 (25)	130 (23)

**Median first recorded hemoglobin (g/L) prior to arrival or in the ED^f ^(IQR^b^)**	123 (109-138)	135 (120-149)

**Lowest documented hemoglobin (g/L) during admission (IQR^b^)**	73 (66-90.50)	92 (76-113)

**Median volume of crystalloid in first 4 hours (ml) (IQRb)**	1950 (950-3675)	1225 (500 - 2403)

**Median hospital length of stay (days)**	17 (8-33)	9 (6-16)

Patients discharged alive (IQR^b^)	17 (8-33)	9 (9-16)

Patients who died during admission (IQR^b^)	16 (4-19)	2 (0-16)

^a ^NOM = non-operative management

^b ^IQR = interquartile range (25th-75th percentile)

^d ^SD = Standard deviation

^e ^BP = Blood Pressure

^f ^ED = Emergency Department

^g ^PRBC = packed red blood cells

According to the a prior definition, there were seven cases of delayed splenic rupture, four occurring during their time in hospital and three occurring in the post-discharge period. All four of patients who had late rupture during their initial admission had splenectomies more than four hours following admission and are included in the failure of SNOM group. Two of these patients presented to the ER late; one week following MVC and two months following an assault. One patient presented five days following injury with splenic rupture (although he was seen in the ER with chest pain one day following injury, observed overnight and discharged home). One patient had a normal CT at the time of admission but after 11 days had become hemodynamically unstable and repeat CT scan showed splenic rupture (although the initial CT scan showed no active extravasation).

The causes of death for these SNOM patients were; nine multiple blunt injuries, five massive head injuries and one case of multiple organ failure. For the operatively managed patients who died during admission the causes of death were: 26 multiple blunt injuries, six massive head injuries/brain deaths, two ruptured aortas, one cerebrovascular accident, one death resulting from complications of sepsis and one death due to exanguination from torn abdominal and retroperitoneal vessels.

Of patients who reside in Calgary (n = 179), three patients who had SNOM were readmitted in the six-month post-discharge period with delayed splenic rupture five, 24 and 26 days following initial injuries. Two of these patients were readmitted for SAA with proximal coil SAE and the third received a splenectomy.

### Splenic Arterial Angiography (SAA) and Embolization (SAE)

**Figure 3:**
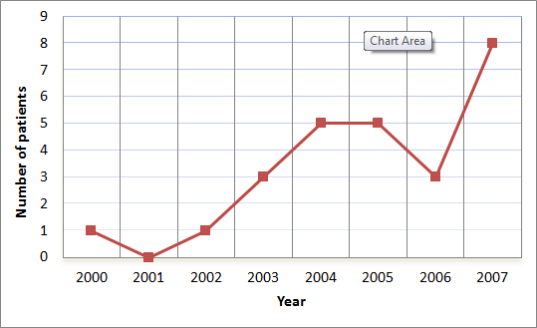
**Utilization of splenic artery angiography (SAA) in patients undergoing nonoperative management (NOM) for acute splenic injury at the Foothills Medical Centre 2000-2007**.

Thirty nine patients (7%) underwent SAA: six from the OM group (4%) and 33 from the SNOM group (9%). Although only a small proportion of the SNOM patients received SAA, the proportion increased over the later years of the study (Figure 3). The need for SAA was at the discretion of the trauma management team. Their age and sex distributions were similar to those of the entire study population (data not shown). Thirty-one patients received some type of SAE, with proximal embolization being the most common procedure (25 patients). The time from arrival to SAA was widely variable (median 15.8 hours, IQR 4.3-73.8). Six SAA patients in the SNOM had documented complications (two with splenic infarct, one case of sepsis four days post-SAA, one case of fever within 48 hrs, one with a follow up CT showing increased peri-splenic hematoma and one persistently hemodynamically unstable patient who died within 24 hours of SAA). One patient in the SNOM group, who had a splenic infarct after SAA, went on to have a diaphragmatic hernia repair but no surgical splenic intervention. No other SNOM patients who received SAA had abdominal surgeries.

Six patients in the OM group had SAA as adjunct therapy. For all six SAA was done post-operatively. Two of these patients were hemodynamically unstable throughout SAA, had multiple extensive injuries and died within 24 hours (one received SAE and one did not). Two patients developed fever within 48 hours of SAA (one received SAE and one did not). One patient received SAE post-operatively with no documented complications.

Overall nine patients (33%) who received SAE had documented complications, but none required operative intervention. Two patients were not at our trauma centre for the entire follow-up period when monitoring for complications was completed. One patient left against medical advice after 24 hours and one patient was transferred to another institution after two days. Both of these patients had multiple small pseudoaneurysms, received proximal SAE with coils and had no documented complications during their admissions.

### Follow-up Imaging in SNOM patients

In our subset analysis of SNOM patients with an admission abdominal CT imaging available for grading and comparison (total 256 patients), overall 50% (129 patients) received follow-up imaging during their hospital admission. We found that patients with higher grade injuries were more likely to receive follow up imaging during their hospital admission with grade 4 or 5 splenic injuries (n = 52) receiving follow up imaging during their hospital admission 56% of the time vs 47% of grade 1 or 2 splenic injuries (n = 151). The indications for abdominal CT imaging were not considered in our study.

The rate of follow-up abdominal imaging in the six-month post-discharge period and readmission to hospital was determined for Calgary residents (Table 2). Thirty six patients (20%) managed by SNOM received post-discharge follow-up imaging. Two of 57 (4%) of operatively managed patients and three of 179 (2%) non-operatively managed patients who were readmitted to hospital. While the small numbers defy formal statistical comparison, all of the SNOM patients readmitted required an intervention (surgery or angiography) while none of the OM group did (Table 2). The two OM patients who were readmitted were for post-splenorraphy hemorrhage not requiring operative intervention and for post-splenectomy sepsis. They were discharged home after seven and five days in hospital respectively in their repeat admissions. Two percent (2%) of SNOM patients were re-admitted to hospital after discharge, with two undergoing SAA with proximal coil SAE, and a third requiring splenectomy. One 40 year old female was initially admitted for three days but required readmission after discharge with SAA and proximal coil SAE. Two patients had late failure of SNOM more than three weeks following their initial injuries. These patients, a 52 year old female and 34 year old male both spent four days in hospital initially and presented to the emergency room with abdominal pain 20 and 22 days following discharge. They required SAA with proximal coil SAE and a splenectomy respectively.

**Table 2:** Imaging and hospital readmissions occurring during the six-month period following discharge for patients residing in Calgary

Outcome	All, N = 236 n (%)	OM^a^, N = 57 n (%)	NOM^b^, N = 179 n (%)
**Abdominal imaging within 6 months of hospital discharge**			

Received follow up CT^c^	41 (17)	5 (9)	36 (20)

No imaging	195 (83)	52 (91)	143 (80)

US^d ^or MRI^e ^only †	2(1)	1 (2)	1 (2)

**Hospital re-admissions and surgeries relating to the spleen injury within 6 months of hospital discharge**			

Re-admitted to hospital	5 (2)	2 (4)	3 (2)

Underwent spleen surgery	0 (0)	0 (0)	1 (1)

Received SAA^f^	2 (1)	0(0)	2 (1)

†One patient in the NOM group had SAA during initial admission and received an abdominal MRI in the follow up period.

^a ^OM = operative management

^b ^NOM = non-operative management

^c ^CT = Computed tomography

^d ^US = ultrasound

^e ^MRI = magnetic resonance imaging

^f ^SAA = Splenic Artery Angiography

## Discussion

SNOM of the hemodynamically stable patient with splenic injury is widely accepted as the current standard of care. At our level 1 trauma centre, the proportion of severely injured trauma patients managed non-operatively increased over time, from 50% in 1996 to 78% in 2007. This finding is consistent with other published studies [22,23]. It has been suggested that improved imaging and detection of splenic injuries has led to a decrease in the severity of splenic injuries that present to trauma centres over time [24,25] which may account for an increase in the use and success of SNOM. We did not observe any trend in injury severity, with median ISS and maximum abdominal injury scores generally stable over time. We did however, observe an increasing proportion of patients receiving SAA over time which may, in part, account for the shift to SNOM as use of adjuvant modalities allows for continued SNOM in patients with ongoing hemorrhage [26].

The overall success rate of SNOM was 87% and associated mortality rate was 4%. Both these success and mortality rates are similar to other published series [6,25,27,28]. Cocanour and colleagues (2000) had a similar patient population with a mean ISS of 20.5 ± 1.1 for all patients with blunt splenic injury (vs. a median ISS of 27 in our population). Their SNOM success rate was also 86% (for 198 patients undergoing SNOM) and the mortality rate was 5%. This study defined a failure of SNOM as requiring splenectomy or splenorrhaphy more than four hours after admission (as we did) but excluded patients who died within 24 hours of admission from massive injuries. Haan and colleagues (2005) found a higher (90%) success rate in SNOM. Their higher success rate may be because of lower overall severity of injuries in their patient population (overall ISS for the 648 patients in Haan and colleagues (2005) was 17 vs our overall ISS of 27) and their higher use of direct OM in their patient group (43% vs only 24% of our patients receiving OM). This difference in success may also relate to our lower than expected rate of adjunctive therapies. Approximately 20% of the patient population in Haan and colleagues (2005) received splenic artery embolization. Although only a small proportion of our SNOM patients were treated with SAA/SAE however, this proportion increased over time, a trend that we expect to continue, as SAA/SAE becomes more accessible to more trauma patients with the advent of interventional/trauma operating/resuscitation rooms [29].

Overall, approximately one third of SAA patient experienced complications (none requiring operative intervention). This is comparable to the rates of major complication (20% to 30%) reported in the literature [5,27,30-32]. Despite the widespread adoption of SNOM, patient selection criteria and the specifics of SNOM treatment protocols are not consistent between institutions [27,33,34]. Without a formal instiutional protocol, the decision to pursue SNOM is left to the discretion of the attending physician. Thus, the reported use and success rates of SNOM strategies for blunt spleen trauma are highly variable. Reviewing the CT findings of our early (< 48 hours) SNOM failures revealed six cases of seemingly unaddressed contrast extravasation or presumed traumatic pseudoaneurysm in otherwise hemodynamically stable patients who later required OM in the earlier years of our review. Although unproven, it is conceivable that early SAA/SAE may have obviated the need for OM, as it might have for the four patients who developed delayed extravasation more than 48 hours after hospital admission. This rationale has led to our introduction of education initiatives and a regional guideline for the management of splenic trauma which includes recommendations regarding addressing these findings in otherwise stable patients [Additional file 2 and Additional file 3].

As hemodynamic instability is the major factor in the decision to opt for OM of splenic injuries, we collected data on fluid and blood requirements during the first four hours of admission. We believe this was an appropriate proxy for the interval during which the decision is typically made to pursue SNOM or OM. As expected, there was a greater need for RBCs and fluid amongst those who received OM, likely due to the greater severity of their injuries. Balaa and colleagues (2004) [8] found that patients who failed SNOM did not require more blood overall than those who received OM initially, however, only 5 of 65 patients in their study failed SNOM. In our study, the 53 patients who failed SNOM required less RBCs and crystalloid fluids than those initially selected for OM, yet more than those who underwent successful SNOM. Demographically, the group of individuals who failed SNOM were slightly older with a median age of 38 (IQR, 25-52) than the successful SNOM patients. This is in agreement with previous studies [25].

Generally, failure of SNOM is reported to occur within the first 48 hours [33,35,36], and we encountered several cases of failure only hours into the hospital admission. These very early failures preclude making comprehensive recommendations regarding early screening for vascular injuries or extravasation amenable to percutaneous therapies, other than to mandate vigilant monitoring and to ensure that an immediate surgical or combined surgical/angiographic response is available. Even if these early failures can be averted however, late failure can occur several days and even weeks or months following injury [19,35]. Of the seven cases of late failure that we experienced, five patients required splenectomy and two were managed with SAA and proximal coil SAE. Our seven cases of delayed splenic rupture occurred five days to two months following the initial injury, four during their hospitalization and three after hospital discharge. Whether vascular lesions pre-disposing to late failure were present in these late failures however, is uncertain due to our limited use of follow-up imaging prior to discharge.

Thus 2% percent of our SNOM patients residing within Calgary were readmitted within 6 months with a delayed splenic complication, and all required either a splenectomy or angioembolization. This rate of late failure of SNOM is particularly concerning given the trend toward early discharge from hospital. These concerns are corroborated by another study by Zarzaur and colleagues (2009) [19] who examined Tennessee hospital readmission data for blunt spleen trauma patients and found that 1.4% of patients (n = 1932) who received SNOM and were discharged home, were readmitted for splenectomy within six months of discharge. Other small case series have described results consistent with our readmission and surgical intervention rate [16,37].

The need for routine follow up imaging for blunt spleen trauma has been widely debated. We found that patients with higher grade injuries were more likely to receive follow-up imaging during their hospital admission. In our subset analysis of SNOM patients with abdominal CT imaging available for grading (total 256 patients), overall 50% (129 patients) received follow-up imaging during their hospital admission. There is good evidence to suggest that follow-up imaging in the two to five days following injury is valuable for detection of splenic pseudoaneurysm [11,13,17]. In our institution we now recommend that repeat CT imaging is obtained within 72 hours in all grades of splenic injury, based on reports documenting the detection of splenic artery pseudoaneurysms even in low Grade injuries [12]. Our protocol also permits the use of dedicated ultrasound exams in selected young patients Grade I and II injuries instead of CT scan based on clinician judgment. It is questionable whether the cost and stochastic risks associated with late abdominal CT imaging is warranted in stable, SNOM patients after discharge from hospital when imaging typically confirms resolution of injury. Other studies have found that CTs taken more than ten days following presentation are generally not used to determine patient treatment or add clinically relevant information [38-40]. Thaemert and colleagues (1997) [14] found that improvement in the appearance of the spleen was evident in all 33 follow up CT scans taken more than 10 days following the injury. Post-discharge imaging was obtained less frequently among our patients (about 17%), with the majority of results confirming improvement or resolution of the spleen injury. Interestingly, SAA patients are overrepresented amongst those who received imaging post-discharge. Eight of the patients in our cohort who received follow-up CTs during the post-discharge period had received SAA on their initial admission. Post-discharge follow-up imaging has been advocated in certain patients to guide return to certain activities and at our institution this is left to the discretion of the attending surgeon. Of the three patients who experienced late-failure in the post-discharge period, all late failure splenic ruptures were detected following onset of patient symptoms. Nevertheless, late follow up imaging may be of value in patients considering return to contact sports or other activities where they may be at risk of re-injury.

The retrospective nature of the data is the greatest limitation of our study. As the decision to treat operatively or not was rarely explicitly noted in the medical record, the definition of what did and did not constitute a decision to treat a patient non-operatively was debatable (i.e., if no surgery within four hours) and based on commonly reported literature. Some patients requiring surgery within this interval may have been selected for SNOM but experienced an unexpected deterioration leading to operative intervention. Alternatively, some patients who went to the operating room outside of the four hour window may have been receiving stabilizing treatment for other injuries, with spleen surgery planned as soon as possible. Accordingly, our estimates of the use of SNOM may not be completely precise. However, it is unlikely that such misclassification was differential over time, and the trend towards increased use of SNOM over time is likely valid. Furthermore, our results may not be generalizable to patients with less severe injuries or patients with isolated spleen injuries since the Alberta Trauma Registry identifies all trauma patients with injury severity scores greater than 12.

At our level 1 trauma centre, the majority of severely injured patients with blunt splenic injuries are currently managed non-operatively with an overall success rate approximating 87%. The low utilization of SAA/SAE potentially offers opportunities to improve this rate in the future. The majority of SNOM failures occur within 24 hours of hospital admission, however, we did observe seven cases of delayed splenic rupture requiring intervention. Although low, rate of delayed splenic rupture and risk of complications resulting from these occurrences suggests that the need for patient education around symptoms of delayed splenic rupture may prove to be increasingly important in our move toward early discharges.

## Abbreviations

AISS: Abbreviated Injury Severity Score; ATV: All-terrain Vehicle; CT: Computed Tomography; DPL: Diagnostic Peritoneal Lavage; ED: Emergency Department FAST: Focused Assessment with Sonography for Trauma; GCS: Glasgow Coma Scale; ICD-9-CM: International Classification of Diseases 9^th ^Revision, Clinical Modification; ICU: Intensive Care Unit; ISS: Injury Severity Score; US: Ultrasound; MRI: Magnetic Resonance Imaging; MVC: Motor Vehicle Collison; NOM: Nonoperative Management; OM: Operative Management; PRBC: Packed Red blood cells; SAE: Splenic Artery Embolization; SBP: Systolic blood pressure.

## Competing interests

The authors declare that they have no competing interests.

## Authors' contributions

Study concept and design: AC; Acquisition of data: DA, AC, AK; Analysis and interpretation of data: AC, CT, CB; Drafting of the manuscript: AC; Critical revision of the manuscript: AC, AK, CB; Study supervision: AK. All authors read and approved the final manuscript.

## Supplementary Material

Additional file 1 **Demographic, clinical and injury characteristics among trauma patients with blunt spleen injury (1996-2007)**. http://www.traumacanada.ca/media/blunt_spleen/File%201%20Supplementary%20Files.pdf. Click here for file

Additional file 2 **Regional trauma services protocol for the management of blunt splenic (April 2007) **[41-51]. http://www.traumacanada.ca/media/blunt_spleen/mgmt_blunt_splenic_trauma.pdf. Click here for file

Additional file 3 **Splenic injury management flowchart**. http://www.traumacanada.ca/media/blunt_spleen/splenic_inj_protocol.pdf. Click here for file

## References

[B1] Brasel KJ DeLisle CM Olson CJ Borgstrom DC Splenic injury: trends in evaluation and management J Trauma-Injury Infect Crit Care 1998 44 283 286 10.1097/00005373-199802000-00006 9498498

[B2] Peitzman AB Heil B Rivera L Federle MB Harbrecht BG Clancy KD Croce M Enderson BL Morris JA Shatz D Blunt selenic injury in adults: multi-institutional study of the Eastern association for the surgery of trauma J Trauma-Injury Infect Crit Care 2000 49 177 187 10.1097/00005373-200008000-00002 10963527

[B3] Nix JA Costanza M Daley BJ Powell MA Enderson BL Outcome of the current management of splenic injuries J Trauma-Injury Infect Crit Care 2001 50 835 841 10.1097/00005373-200105000-00010 11371838

[B4] Dent D Alsabrook G Erickson BA Myers J Wholey M Stewart R Root H Ferral H Postoak D Napier D Pruitt BA Blunt splenic injuries: High nonoperative management rate can be achieved with selective embolization J Trauma-Injury Infect Crit Care 2004 56 1063 1067 10.1097/01.TA.0000123037.66867.F2 15179247

[B5] Haan JM Biffl W Knudson MM Davis KA Oka T Majercik S Dicker R Marder S Scalea TM Splenic embolization revisited: a multicenter review J Trauma-Injury Infect Crit Care 2004 56 542 547 10.1097/01.TA.0000114069.73054.45 15128125

[B6] Haan JM Bochicchio GV Kramer N Scalea TM Nonoperative management of blunt splenic injury: A 5-year experience J Trauma-Injury Infect Crit Care 2005 58 492 498 10.1097/01.TA.0000154575.49388.74 15761342

[B7] Smith JS Cooney RN Mucha P Nonoperative management of the ruptured spleen: a revalidation of criteria Surgery 1996 120 745 750 10.1016/S0039-6060(96)80026-2 8862387

[B8] Balaa F Yelle JD Pagliarello G Lorimer J O'Brien JA Isolated blunt splenic injury: Do we transfuse more in an attempt to operate less? Can J Surg 2004 47 446 450 15646444 PMC3211584

[B9] Cocanour CS Moore FA Ware DN Marvin RG Clark JM Duke JH Delayed complications of nonoperative management of blunt adult splenic trauma Arch Surg 1998 133 619 624 10.1001/archsurg.133.6.619 9637460

[B10] Haan JM Marmery H Shanmuganathan K Mirvis SE Scalea TM Experience with splenic main coil embolization and significance of new or persistent pseudoaneurym: reembolize, operate, or observe J Trauma-Injury Infect Crit Care 2007 63 615 619 10.1097/TA.0b013e318142d244 18073609

[B11] Weinberg JA Magnotti LJ Croce MA Edwards NM Fabian TC The utility of serial computed tomography imaging of blunt splenic injury: Still worth a second look? J Trauma-Injury Infect Crit Care 2007 62 1143 1147 10.1097/TA.0b013e318047b7c2 17495714

[B12] Weinberg JA Lockhart ME Parmar AD Griffin RL Melton SM Vandromme MJ McGwin G Rue LW Computed tomography identification of latent Pseudoaneurysm after blunt splenic injury: pathology or technology? J Trauma-Injury Infect Crit Care 2010 68 1112 1116 10.1097/TA.0b013e3181d769fc 20453766

[B13] Schroeppel TJ Croce MA Diagnosis and management of blunt abdominal solid organ injury Curr Opin Crit Care 2007 13 399 404 10.1097/MCC.0b013e32825a6a32 17599009

[B14] Thaemert BC Cogbill TH Lambert PJ Nonoperative management of splenic injury: are follow-up computed tomographic scans of any value? J Trauma-Injury Infect Crit Care 1997 43 748 751 10.1097/00005373-199711000-00003 9390484

[B15] Peitzman AB Harbrecht BG Rivera L Heil B Eastern Assoc Surg Trauma M: failure of observation of blunt splenic injury in adults: Variability in practice and adverse consequences J Am Coll Surg 2005 201 179 187 10.1016/j.jamcollsurg.2005.03.037 16038813

[B16] Crawford RS Tabbara M Sheridan R Spaniolas K Velmahos GC Early discharge after nonoperative management for splenic injuries: increased patient risk caused by late failure? Surgery 2007 142 337 342 10.1016/j.surg.2007.05.003 17723884

[B17] Smith J Armen S Cook CH Martin LC Blunt splenic injuries: have we watched long enough? J Trauma-Injury Infect Crit Care 2008 64 656 663 10.1097/TA.0b013e3181650fb4 18332805

[B18] Cocanour CS Moore FA Ware DN Marvin RG Duke JH Age should not be a consideration for nonoperative management of blunt splenic injury J Trauma-Injury Infect Crit Care 2000 48 606 610 10.1097/00005373-200004000-00005 10780591

[B19] Zarzaur BL Vashi S Magnotti LJ Croce MA Fabian TC The real risk of splenectomy after discharge home following nonoperative management of blunt splenic injury J Trauma-Injury Infect Crit Care 2009 66 1531 1538 10.1097/TA.0b013e3181a4ed11 19509611

[B20] Association for the Advancement of Automotive Medicine Committee on Injury Scaling The Abbreviated Injury Scale--1998 Revision (AIS-98) 1998 Medicine. AftAoA ed. Des Plaines, IL.

[B21] National Center for Health Statistics, International Classification of Diseases, 9th revision, Clinical Modification (ICD-9-CM) http://www.cdc.gov/nchs/icd/icd9cm.htm

[B22] Cadeddu M Garnett A Al-Anezi K Farrokhyar F Management of spleen injuries in the adult trauma population: a ten-year experience Can J Surg 2006 49 386 390 17234065 PMC3207549

[B23] Haan J Scott J Boyd-Kranis RL Kramer M Scalea TM Admission angiography for blunt splenic injury: advantages and pitfalls J Trauma-Injury Infect Crit Care 2001 51 1161 1165 10.1097/00005373-200112000-00023 11740269

[B24] Harbrecht BG Zenati MS Ochoa JB Puyana JC Alarcon LH Peitzman AB Evaluation of a 15-year experience with splenic injuries in a state trauma system Surgery 2007 141 229 238 10.1016/j.surg.2006.06.032 17263980

[B25] Harbrecht BG Ko SH Watson GA Forsythe RM Rosengart MR Peitzman AB Angiography for blunt splenic trauma does not improve the success rate of nonoperative management J Trauma-Injury Infect Crit Care 2007 63 44 49 10.1097/TA.0b013e3180686531 17622867

[B26] Liu PP Lee WC Cheng YF Hsieh PM Hsieh YM Tan BL Chen FC Huang TC Tung CC Use of splenic artery embolization as an adjunct to nonsurgical management of blunt splenic injury J Trauma-Injury Infect Crit Care 2004 56 768 772 10.1097/01.TA.0000129646.14777.ff 15187739

[B27] Cooney R Ku J Cherry R Maish GO Carney D Scorza LB Smith JS Limitations of splenic angioembolization in treating blunt splenic injury J Trauma-Injury Infect Crit Care 2005 59 926 932 10.1097/01.ta.0000188134.32106.89 16374283

[B28] Smith HE Biffl WL Majercik SD Jednacz J Lambiase R Cioffi WG Splenic artery embolization: have we gone too far? J Trauma-Injury Infect Crit Care 2006 61 541 544 10.1097/01.ta.0000235920.92385.2b 16966984

[B29] Ball C Kirkpatrick A D'Amours S The RAPTOR: resuscitation with angiography, percutaneous techniques and operative repair. Transforming the discipline of trauma surgery Canadian J Surgery 2011 54 E3 E4 PMC3195651 21933518

[B30] Sclafani SJA Shaftan GW Scalea TM Patterson LA Kohl L Kantor A Herskowitz MM Hoffer EK Henry S Dresner LS Wetzel W Nonoperative salvage of computed Tomography-diagnosed splenic injuries - utilization of angiography for triage and embolization for hemostasis J Trauma-Injury Infect Crit Care 1995 39 818 827 10.1097/00005373-199511000-00004 7473996

[B31] Wu SC Chen RJ Yang AD Tung CC Lee KH Complications associated with embolization in the treatment of blunt splenic injury World J Surgery 2008 32 476 482 10.1007/s00268-007-9322-x 18175174

[B32] Ekeh AP McCarthy MC Woods RJ Haley E Complications arising from splenic embolization after blunt splenic trauma Am J Surg 2005 189 335 339 10.1016/j.amjsurg.2004.11.033 15792763

[B33] Velmahos GC Toutouzas KG Radin R Chan L Demetriades D Nonoperative treatment of blunt injury to solid abdominal organs - A prospective study Arch Surg 2003 138 844 849 10.1001/archsurg.138.8.844 12912742

[B34] Wu SC Chow KC Lee KH Tung CC Yang AD Lo CJ Early selective angioembolization improves success of nonoperative management of blunt splenic injury Am Surg 2007 73 897 902 17939422

[B35] Bala M Edden Y Mintz Y Kisselgoff D Gercenstein I Rivkind AI Farugy M Almogy G Blunt splenic trauma: predictors for successful non-operative management Israel Med Assoc J 2007 9 857 861 18210925

[B36] Meguid AA Bair HA Howells GA Bendick PJ Kerr HH Villalba MR Prospective evaluation of criteria for the nonoperative management of blunt splenic trauma Am Surg 2003 69 238 242 12678481

[B37] Savage SA Zarzaur BL Magnotti LJ Weinberg JA Maish GO Bee TK Minard G Schroeppel T Croce MA Fabian TC The evolution of blunt splenic injury: resolution and progression J Trauma-Injury Infect Crit Care 2008 64 1085 1091 10.1097/TA.0b013e31816920f1 18404079

[B38] Schurr MJ Fabian TC Gavant M Croce MA Kudsk KA Minard G Woodman G Pritchard FE Management of blunt splenic trauma - computed tomographic contrast blush predicts failure of nonoperative management J Trauma-Injury Infect Crit Care 1995 39 507 513 10.1097/00005373-199509000-00018 7473916

[B39] Lawson DE Jacobson JA Spizarny DL Pranikoff T Splenic trauma - value of follow-up ct Radiology 1995 194 97 100 7997589 10.1148/radiology.194.1.7997589

[B40] Norrman G Tingstedt B Ekelund M Andersson R Nonoperative management of blunt splenic trauma: also feasible and safe in centers with low trauma incidence and in the presence of established risk factors Eur J Trauma Emergency Surgery 2009 35 102 107 10.1007/s00068-008-8108-7 26814761

[B41] Trunkey DD Trauma Sci Am 1983 249 28 35 10.1038/scientificamerican0883-28 6623052

[B42] Houtchens BA Major trauma in the rural mountain west Ann Emerg Med 1977 6 343 50 10.1016/s0361-1124(77)80167-6 886667

[B43] Peitzman AB Heil B Rivera L Federle MB Harbrecht BG Clancy KD Blunt splenic injury in adults: Multi-institutional study of the Eastern Association for the Surgery of Trauma J Trauma 2000 49 177 189 10.1097/00005373-200008000-00002 10963527

[B44] Haan J Scott J Boyd-Kranis RL Ho S Kramer M Scalea TM Admission angiography for blunt splenic injury: advantages and pitfalls J Trauma 2001 51 1161 1165 10.1097/00005373-200112000-00023 11740269

[B45] Brasel K Delisle C Olson C Borgstrom O Splenic injury: trends in evaluation and management J Trauma 1998 44 283 285 10.1097/00005373-199802000-00006 9498498

[B46] Kirkpatrick AW Clinician-performed focused sonography for the resuscitation of trauma Crit Care Med 2007 35 S162 S172 10.1097/01.CCM.0000260627.97284.5D 17446775

[B47] Kirkpatrick AW Sirois M Laupland KB Goldstein L Brown DR Simons RK The hand-held FAST exam for blunt trauma Can J Surg 2005 48 453 460 16417051 PMC3211725

[B48] Moore EE Cogbill TH Jurkovich GJ Shackford SR Malagoni MA Champion HR Organ injury scaling: spleen and liver (1994 revision) J Trauma 1995 38 323 324 10.1097/00005373-199503000-00001 7897707

[B49] Haan JM Bochicchio GV Kramer N Scalea TM Nonoperative management of blunt splenic injury: a 5-year experience J Trauma 2005 58 492 498 10.1097/01.TA.0000154575.49388.74 15761342

[B50] Haan JM Boswell S Stein D Scalea TM Follow-up abdominal CT is not necessary in low-grade splenic injury Am Surg 2007 73 13 18 17249449

[B51] Weinberg JA Magnotti LJ Croce MA Edwards NM Fabian TC The utility of serial computed tomography imaging of blunt splenic injury: Still worth a second look? J Trauma 2007 62 1143 1148 10.1097/TA.0b013e318047b7c2 17495714

