# Supplementary material for: The song remains the same although the instruments are changing: complications following selective non-operative management of blunt spleen trauma: a retrospective review of patients at a level I trauma centre from 1996 to 2007
Source: J Trauma Manag Outcomes. 2012 Mar 13;6:4. doi: 10.1186/1752-2897-6-4 (PMC3338082; doi:10.1186/1752-2897-6-4)
Supplement: Additional file 1 — Demographic, clinical and injury characteristics among trauma patients with blunt spleen injury (1996-2007). http://www.traumacanada.ca/media/blunt_spleen/File%201%20Supplementary%20Files.pdf. [file 1752-2897-6-4-S1.PDF]

On-line Supplementary File 1. Demographic, clinical and injury characteristics among trauma patients with blunt spleen injury (1996-2007).

Characteristic	**All, N=538, n (%)**	**OM[a], N=150, n (%)**	**NOM[b], N=388, n (%)**
**Place of residence**			
Calgary	261 (49)	79 (53)	182 (47)
Other	277 (51)	71 (47)	206 (53)
**Median age (IQR)[c]**	34 (23-49)	33 (23-46.5)	34 (23-49)
**Sex**			
Male	395 (73)	117 (78)	278 (72)
Female	143 (27)	33 (22)	110 (28)
**Injury severity score (ISS)**			
Median ISS (IQR)[c]	27 (19-34)	34 (25 – 43)	24 (17 -32)
ISS < 25	231 (43)	30 (20)	201 (52)
ISS 25+	307 (57)	120 (80)	187 (48)
**MAIS[f] head**			
No head injury	244 (45)	62 (41)	182 (47)
1-2	113 (21)	20 (13)	93 (24)
3-5	180 (33)	67 (45)	113 (29)
6	1 (1)	1 (1)	0 (0)
**MAIS abdomen**			
1-2	250 (46)	29 (19)	221 (57)
3-5	288 (54)	121 (81)	167 (43)
**Hours between injury event and arrival at trauma centre**			
<1	159 (30)	54 (36)	105 (27)
1-4	185 (34)	56 (38)	129 (33)
4-24	133 (25)	24 (16)	109 (28)
> 24	14 (3)	2 (1)	12 (3)
Not documented	47 (9)	14 (9)	33 (8)
Median (IQR[c])	1.95 (0.80 – 4.55)	1.63 (0.58 – 3.47)	2.17 (0.88 – 5.25)
**Interventions required prior to arrival or in the emergency department**			
**Endotracheal intubation**	194 (36)	93 (62)	101 (26)
**Assisted ventilation**	182 (34)	90 (60)	92 (24)
**CPR, defibrillation or conversion**	20 (4)	17 (11)	3 (1)
**First recorded GCS[d]**			
3-8	20 (4)	12 (8)	8 (2)
9-12	10 (2)	2 (1)	8 (2)
13-15	335 (62)	72 (48)	263 (68)
Unknown / unable to record/missing	173 (32)	64 (43)	109 (28)
**Fluid resuscitation requirements in the first 4 hours**			
Median units PRBC[j](IQR[c])7 missing	0 (0-2)	3 (1-7)	0 (0-0)

Median volume of crystalloid (ml) (IQR[c])5 missing	2000 (825 - 4000)	5400 (3000 – 7500)	1300 (500 -2800)

[a]OM = operative management
[b] NOM = non-operative management
[c] IQR = interquartile range
[d] ATV = All-Terrain Vehicle
[e] Other includes: 3 railway incidents, 1 parachute incident, 1 avalanche victim, 2 explosions and 2 patients crushed between objects
[f]MAIS = maximum abbreviated injury score
