# Supplementary material for: The song remains the same although the instruments are changing: complications following selective non-operative management of blunt spleen trauma: a retrospective review of patients at a level I trauma centre from 1996 to 2007
Source: J Trauma Manag Outcomes. 2012 Mar 13;6:4. doi: 10.1186/1752-2897-6-4 (PMC3338082; doi:10.1186/1752-2897-6-4)
Supplement: Additional file 2 — Regional trauma services protocol for the management of blunt splenic (April 2007) [41-51]. http://www.traumacanada.ca/media/blunt_spleen/mgmt_blunt_splenic_trauma.pdf. [file 1752-2897-6-4-S2.PDF]

Subject/Title: **Management of Blunt Splenic Injuries**	Reference Number: **U - 600**	
	Date Established: 2007-04: Draft	
Authorization: Medical Director: Trauma Services	Date Revised:	Date Reviewed:
	Page: 1 of 2	

This guideline is intended to serve as an institutional resource to guide general management of the patient with an injured spleen. It is not meant to be a substitute for good clinical judgment, appropriate consultation, and close clinical follow-up.

POINTS OF EMPHASIS

The spleen is one of the most commonly injured intra-peritoneal injuries, especially in the major trauma population admitted to the Foothills Medical Centre. Untreated or poorly treated splenic injuries are a major source of preventable morbidity and mortality[1,2]. Mortality from hemorrhagic shock due to splenic bleeding is most expeditiously treated with either a splenic salvage operation or more commonly a splenectomy. The optimal management of blunt splenic injury has undergone a dramatic evolution over the past several decades though, largely due to the recognition of the innate healing potential of the spleen and improvements in computed tomography. Non-operative management of blunt splenic injury in those who are hemodynamically stable is currently the standard of care[3-5]. Although the vast majority of those with blunt splenic injuries managed in this way, do well and are able to keep their spleens, there is now a minority of patients who fail non-operative management due to delayed vascular complications that were never seen when splenectomy was the standard approach to splenic injury. Controversy and practice variation exists over when and in whom to perform studies aimed at detecting vascular complications of blunt splenic injury.

GUIDELINES

1. Hemodynamically unstable patients (those with overt shock such as a systolic blood pressure < 90 mmHg) should NOT be transported to the CT scan.
2. Hemodynamically unstable patients suspected of having intra-peritoneal bleeding (those with overt shock such as a systolic blood pressure < 90 mmHg) should remain in the trauma room or transported to an operating room for further evaluation and/or therapy[3].
3. Appropriate diagnostic methods for ruling in or out an abdominal source of hemorrhage in those with shock are either a Focused Assessment with Sonography for Trauma (FAST) or a diagnostic peritoneal lavage (DPL)[6,7].
4. Hemodynamically stable patients suspected of having blunt splenic trauma should undergo and abdominal CT scan.
5. Hemodynamically stable (systolic blood pressure > 90 mmHg) with evidence of on-going bleeding detected on CT scanning as evidenced by active extravasation of contrast require

DRAFT

definitive and prompt hemorrhage control through either; a) surgical intervention, or b) angioembolization.

6. All hemodynamically stable patients diagnosed with a splenic injury require admission and close serial observation through;

   a) serial physical examination every six hours

   b) serial hemoglobin level determination every six hours

   c) hourly vital sign monitoring

   until such a time as all the factors have been stable for 24 hours.

7. All splenic injuries should be accurately graded, and documented on the patient's health record, by the attending trauma surgeon using the Organ Injury Scale for Splenic Trauma of the Organ Injury Scaling Committee of the American association for the Surgery of Trauma[8].

8. **All** splenic injuries should undergo repeat scanning 72-96 hours after admission to detect vascular complications. While specific indications for follow-up diagnostic/therapeutic angiography have included but are not limited to[9,10];

   a) Grade III-V scoring

   b) contrast extravasation

   c) pseudoaneurysm

   d) arteriovenous fistula

   e) abrupt vessel truncation

Low grade splenic injuries Grade I – II remain controversial as to their risk of developing psuedoaneurysms. Despite this, one the of best studies to date noted that 24% of all psuedoaneurysms detected were in grade I and II splenic injuries[11], and we have personally observed cases of traumatic pseudoaneurysm in "low grade" splenic injuries in this institution therefore justifying continued screening of all patients in our opinion. In young patients with a low-grade (Grade I – II) injury, a follow-up ultrasound with specified instructions to the ultrasonographer to interrogate the spleen, looking for a pseudoaneurysm *may* be an acceptable practice.

9. Long term follow-up (both clinical and radiologic) of blunt splenic injuries is not addressed by this particular guideline, but is recognized as both a challenge and

DRAFT

research opportunity given the large number of blunt splenic injuries in physically active people wishing an early return to both vocational and recreational activities in the CHR, as well as the large number of national and international visitors to our geographic catchments area requiring repatriation to their homes.

10. It is recognized that in exceptional cases, hemodynamically unstable patients may be taken directly from the trauma room to an interventional angiography suite when a pelvic/retroperitoneal source of bleeding is suspected. Until the hybrid trauma OR (RAPTOR – Resuscitation with Angiographic Percutaneous Treatments and Operative Resuscitation) is functional these cases will be considered exceptional and reflect the on the scene best decision making of the responsible attending Trauma Surgeon.

DRAFT

Appendix:

DRAFT

Subject/Title: **Management of Blunt Splenic Injuries**	Date Established 2007-04	Date Reviewed/ Revised:	Reference Number: U-600	Page: 2 of 2

Grade[a]		Injury Description	ICD-9	AIS-90
I	Hematoma	Subcapsular, <10% surface area	865.01<br>865.11	2
	Laceration	Capsular tear, <1 cm parenchymal depth	865.02<br>865.12	2
II	Hematoma	Subcapsular, 10–50% surface area; intraparenchymal, <5 cm in diameter	865.01<br>865.11	2
	Laceration	1–3 cm parenchymal depth which does not involve a trabecular vessel	865.02<br>865.12	
III	Hematoma	Subcapsular, >50% surface area or expanding; ruptured subcapsular or parenchymal hematoma<br>Intraparenchymal hematoma >5 cm or expanding		3
	Laceration	>3 cm parenchymal depth or involving trabecular vessels	865.03<br>865.13	3
IV	Laceration	Laceration involving segmental or hilar vessels producing major devascularization (>25% of spleen)		4
V	Laceration	Completely shattered spleen	865.04<br>865.14	5
	Vascular	Hilar vascular injury which devascularizes spleen		5

[a] Advance one grade for multiple injuries, up to grade III.

DRAFT

References

Reference List

1. Trunkey DD. Trauma. Sci Am 1983;249:28-35.

2. Houtchens BA. Major trauma in the rural mountain west. Ann Emerg Med 1977;6:343-50.

3. Peitzman AB, Heil B, Rivera L, Federle MB, Harbrecht BG, Clancy KD, et al. Blunt splenic injury in adults: Multi-institutional study of the Eastern Association for the Surgery of Trauma. J Trauma 2000;49:177-89.

4. Haan J, Scott J, Boyd-Kranis RL, Ho S, Kramer M, Scalea TM. Admission angiography for blunt splenic injury: advantages and pitfalls. J Trauma 2001;51:1161-5.

5. Brasel K, Delisle C, Olson C, Borgstrom O. Splenic injury: trends in evaluation and management. J Trauma 1998;44:283-5.

6. Kirkpatrick AW. Clinician-performed focused sonography for the resuscitation of trauma. Crit Care Med 2007;35:S162-S172.

7. Kirkpatrick AW, Sirois M, Laupland KB, Goldstein L, Brown DR, Simons RK, et al. The hand-held FAST exam for blunt trauma. Can J Surg 2005;48:453-60.

8. Moore EE, Cogbill TH, Jurkovich GJ, Shackford SR, Malagoni MA, Champion HR. Organ injury scaling: spleen and liver (1994 revision). J Trauma 1995;38:323-4.

9. Haan JM, Bochicchio GV, Kramer N, Scalea TM. Nonoperative management of blunt splenic injury: a 5-year experience. J Trauma 2005;58:492-8.

10. Haan JM, Boswell S, Stein D, Scalea TM. Follow-up abdominal CT is not necessary in low-grade splenic injury. Am Surg 2007;73:13-8.

11. Weinberg JA, Magnotti LJ, Croce MA, Edwards NM, Fabian TC. The utility of serial computed tomography imaging of blunt splenic injury: Still worth a second look? J Trauma 2007;62:1143-1148.
