# Supplementary material for: The song remains the same although the instruments are changing: complications following selective non-operative management of blunt spleen trauma: a retrospective review of patients at a level I trauma centre from 1996 to 2007
Source: J Trauma Manag Outcomes. 2012 Mar 13;6:4. doi: 10.1186/1752-2897-6-4 (PMC3338082; doi:10.1186/1752-2897-6-4)
Supplement: Additional file 3 — Splenic injury management flowchart. http://www.traumacanada.ca/media/blunt_spleen/splenic_inj_protocol.pdf. [file 1752-2897-6-4-S3.PDF]

June 2009

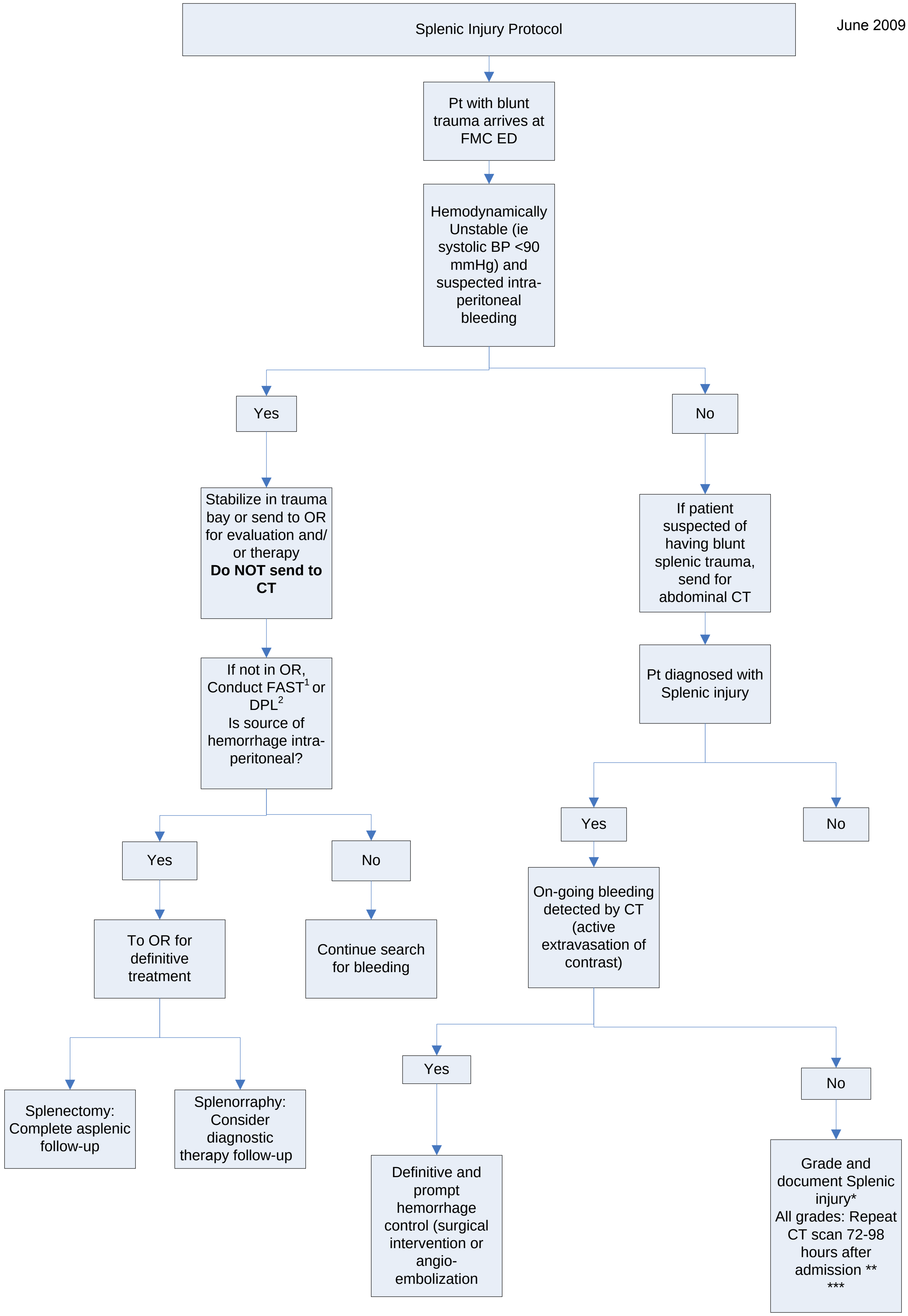

[1] Focused Assessment with Sonography for Trauma
[2] Diagnostic Peritoneal Lavage
*Grade splenic injury using the Organ Injury Scale for Splenic Trauma of the Organ Injury Scaling Committee of the American association for the Surgery of Trauma. Document Grade on Patient Health Record
** All splenic injuries should undergo repeat scanning to detect vascular compromise. Specific indications for f/u diagnostic/therapeutic angiography have included but are not limited to [9.10] : a) Grade III-V scoring; b) contrast extravasation; c) pseudoaneurysm; d) arteriovenous fistula; e) abrupt vessel truncation/
***For Grade I-II splenic injuries, and young of age, may consider follow-up with dedicated splenic ultra-sound looking for pseudoaneurysm
